# Effect of pre-operative neuromuscular training on functional outcome after total knee replacement: a randomized-controlled trial

**DOI:** 10.1186/1471-2474-14-157

**Published:** 2013-05-03

**Authors:** Erika O Huber, Rob A de Bie, Ewa M Roos, Heike A Bischoff-Ferrari

**Affiliations:** 1 Centre on Aging an Mobility, University Hospital Zurich and Waid City Hospital Zurich, University of Zurich, Gloriastrasse 25, Zurich 8091, Switzerland; 2 Institute of Physiotherapy, School of Health Professions, Zurich University of Applied Sciences, Technikumstrasse 71, Postfach, Winterthur 8401, Switzerland; 3 Department of Epidemiology, CAPHRI School of Public Health and Primary Care, Maastricht University, Maastricht, The Netherlands; 4 Institute of Sports Science and Clinical Biomechanics, University of Southern Denmark, Campusvey 55, Odense M, 5230, Denmark

## Abstract

**Background:**

Total Knee Replacement (TKR) is the standard treatment for patients with severe knee osteoarthritis (OA). Significant improvement in pain and function are seen after TKR and approximately 80% of patients are very satisfied with the outcome. Functional status prior to TKR is a major predictor of outcome after the intervention. Thus, improving functional status prior to surgery through exercise may improve after surgery outcome. However, results from several previous trials testing the concept have been inconclusive after surgery.

**Methods/design:**

In a randomized controlled trial (RCT) we will test the effect of a pre-operative neuromuscular trainingprogram versus an attention control program on lower extremity function – before and after surgery. We will enroll 80 participants, aged between 55–90 years, who are scheduled for TKR. In this single-blinded RCT, the intervention group will receive a minimum of 8 and a maximum of 24 training sessions plus 3 educational sessions of the knee school. The control group will receive the 3 educational sessions only. Assessments are performed immediately before and after the intervention (before surgery), at 6 weeks, 3 months and 12 months (after surgery).

The primary outcome will include the Chair Stand Test as a measure of leg strength and reaction time. Secondary outcomes are knee function and pain assessed with the self-reported Knee Injury and Osteoarthritis Outcome Score (KOOS). All measurements will be carried out by a specially trained physical therapist, blinded to group allocation.

**Discussion:**

To our knowledge this is the first single-blinded RCT to test the effect of pre-operative neuromuscular training plus knee school against knee school alone – on knee function and pain, assessed immediately after the interventions prior to surgery and repeatedly after surgery.

**Trial registration:**

Clinical Trials NCT00913575

## Background

Osteoarthritis (OA) is the most common joint disorder and a common cause of pain, loss of function and disability in older adults [1]. It is the second most common diagnosis made in older adults seeking medical care [2] and the leading cause of disability at older age [3]. When suffering from severe OA, Total Joint Replacement (TJR) is the preferred treatment option to significantly improve function and pain [2,4]. Given the demographic change with growing segment of the senior population in the Western World, the rate of these procedures will rise exponentially over the next decade. This will result in high health-care expenditures due to the absolute increase in TJR surgery (both direct hospital charges and indirect costs) [5].

The current EULAR (European League Against Rheumatism) recommendations include exercise as an effective treatment in the improvement of pain and function in patients with moderate to severe knee OA (effect size for validated outcome measures of pain and function versus placebo range from 0.57 to 1.0) [6]. Similarly, the OARSI (Osteoarthritis Research Society International) supports the benefits of exercise in patients with knee OA, both on pain and function [7].

Notably, exercise alone may delay but not prevent TJR in severe OA [8]. Similarly, however, TJR does not fully restore function in many patients undergoing the procedure [9-11], which in part may be due to long-term mechanical impairments of the joint.

The most recent review published on pre-operative interventions for patients with hip or knee osteoarthritis awaiting joint replacement was published in 2011 and included 23 trials [12]. The authors concluded that there was low to moderate quality evidence for the benefit of pre-operative exercise for Total Knee Replacement (TKR). These data are consistent with three earlier systematic reviews [13-15].

Considering the evidence from the available formal reviews, two factors may have prevented a benefit from these programs: (A): Four trials had very small groups with a combined total of 137 subjects and were probably underpowered [16-19]. (B): The therapeutic validity as described by Hoogeboom et al. was not sufficient enough, making it unlikely, that interventions evaluated in these studies would have relevant effects [20].

In this study we aim to test a well-defined and feasible training program plus a knee school educational package against the educational package alone on lower extremity function and pain before and after TKR. The trial is powered based on pilot data and includes a detailed assessment of adherence to the program.

### Objective

To study the effect of a pre-operative neuromuscular training plus knee school educational program compared to the educational program alone on lower extremity function and pain in individuals age 55–90 on a waiting list for TKR due to severe knee OA.

### Hypothesis

Primary Endpoint: We hypothesize that patients undergoing a pre-operative neuromuscular training in addition to the educational program will be significantly quicker in performing the Chair Stand Test (test of lower extremity function) compared with those receiving the educational program alone immediately after the intervention and after TKR surgery.

Secondary Endpoints: We hypothesize that patients undergoing a pre-operative neuromuscular training in addition to the educational program will have a greater improvement in function and pain (KOOS subscales) compared with those receiving the educational program alone immediately after the intervention and after TKR surgery.

## Methods/design

### Design

**Figure 1:**
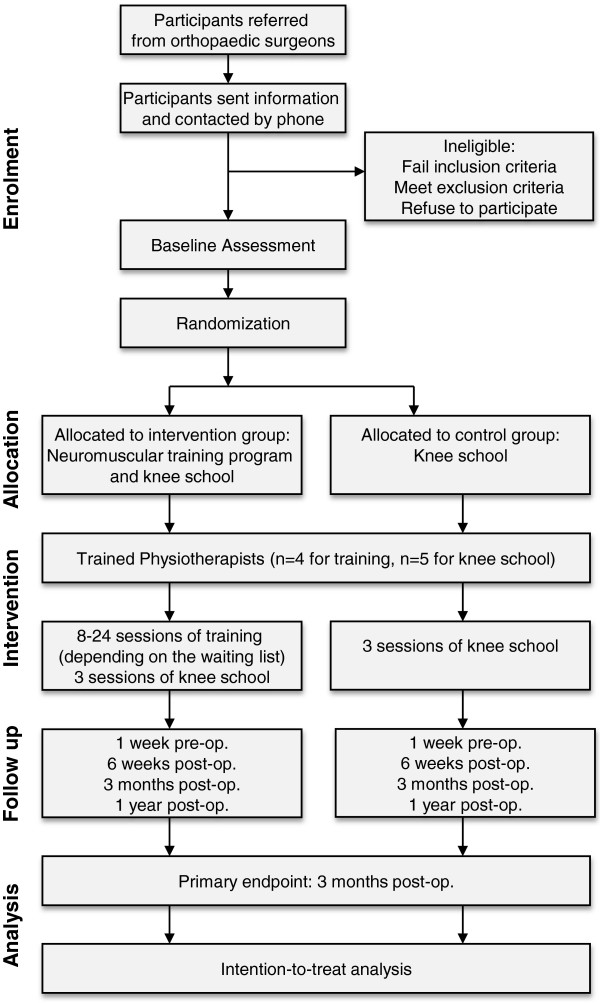
Flow chart of research design.

The study design is a single-blinded randomized controlled trial (Figure 1).

Outcomes are measured at baseline (6–12 weeks pre-operative: primary and secondary endpoints), 1 week pre-operative (after the intervention: primary and secondary endpoints), 6 weeks after surgery (secondary endpoints only), 3 months after surgery (primary and secondary endpoints) and 1 year after surgery (secondary endpoints only).

Prior to the baseline assessment eligible patients receive an information package on the study. The package includes detailed information, including their rights when participating in a research project and a written informed consent form. Ethic approval was granted by the Ethics Committee of the Cantons Aargau and Solothurn, Switzerland, approval number 2009/012.

Reporting of the RCT follows the recommendations of CONSORT guidelines for reporting of clinical trials and will be based on intent-to-treat [21]. In addition, a per-protocol analysis will be performed.

### Participants and recruitment

We include individuals age 55–90 years on a waiting list for TKR due to severe primary or secondary OA. Individuals need to also be community dwelling and understand German as a written and spoken language. Exclusion criteria are cognitive impairment, revision surgery for TKR, plans to leave Switzerland before or after surgery, history of inflammatory arthritis and inability to walk at least 3 meters with or without walking aid.

The trial recruits at the Cantonal Hospital Olten and the Cantonal Hospital Aarau, which both perform about 200 TKRs per year. Recruitment and eligibility assessment are conducted by the orthopaedic surgeon at the time of consultation, when the patient has decided to undergo TKR surgery and is placed on the waiting list. Eligible patients are referred to the study centre (Centre on Aging and Mobility, University of Zurich) by fax. The referring surgeon gives eligible patients a one-page summary of the study. After confirming their interest, eligible individuals receive a detailed participant information about the study procedure and 4–7 days later, they are contacted by phone (EOH) to answer additional questions. Eligible and willing individuals then provide their written informed consent. Two experienced physical therapists (assessors), not working at the recruitment sites and not being involved in the neuromuscular training and the knee school, have been specifically trained for the assessments of this study and are blinded to the randomization.

Neuromuscular training and knee school take place at the recruitment sites. Several experienced physical therapists working at the recruitment sites, have been specially trained either for the neuromuscular training or the knee school. Individuals of the intervention group and individuals of the control group were not together in the knee school.

### Baseline assessment and randomization

After informed consent, the assessor performs the baseline assessment at the recruitment site. Additionally, demographics and covariates that affect outcomes are documented: age, gender, height, weight, cognitive impairment (assessed with the Folstein Mini Mental score) [22] and risk of extended inpatient rehabilitation (measured with the Risk Assessment and Predictor Tool (RAPT) [23].

Participants will be randomized using block allocation with a block size of four. The list is computer generated by the independent randomization centre.

The coordinating investigator (EOH) informs the participant about the group allocation and schedules the treatment sessions as allocated by the independent randomization centre.

### Interventions

#### Neuromuscular training program (NEMEX-TJR training program)

The neuromuscular training follows the principles of neuromuscular and biomechanical training as described in the neuromuscular training method NEuroMuscular EXercise (NEMEX) [24], and aims to improve neuromuscular control (also called sensorimotor control) and to achieve compensatory functional stability. Neuromuscular control is defined as the ability to produce controlled movement through coordinated muscle activity. Movement starts with the non-affected leg to facilitate the bilateral transfer effect of motor learning. Exercises are mainly performed in closed kinetic chains in the lying, sitting or standing position to achieve muscular co-activation in an appropriate position of the joints with hip, knee and foot well aligned. These activities emphasize the improvement of the stabilizing functions of the weight-bearing muscles for better postural control in static and dynamic situations. Open kinetic chains are only performed to improve muscle strength of the knee and hip muscles.

The training takes place in groups under the supervision of an experienced and especially trained physical therapist and consists of three parts:

• Part 1 Warming up: The warm-up period consists of ergometer cycling for 10 minutes. The workload is set individually and can be increased during the 10 minutes.

• Part 2 Circuit program: The circuit program comprises four exercise circles, with the key elements: core stability/postural function, functional alignment, lower-extremity muscle strength and functional exercises. Usually two exercises, sometimes one, are performed in each circle. Each exercise is performed 2–3 sets * 10–15 repetitions, with rest between each exercise and circle. To allow progression three levels of difficulty are defined. Progression is provided by varying in number, direction and velocity of the movements, by increasing the load and/or by changing the support surface. Progression is made when an exercise is performed of 3 sets * 15 repetitions with good neuromuscular control and good quality of performance (based on visual inspection by the physical therapist) and with minimal exertion and control of the movement (perceived by the patient).

• Part 3: Cooling down: The cooling down period of about 10 minutes consists of forward and backward walking exercises, about 10 meters in each direction, mobility exercises for the lower extremities and stretching exercises for the lower-extremity muscles.

The exercises included in the training program are described in detail in the Additional file 1[24].

Documentation of the training program includes the number of training sessions, level of difficulty per session, pain on a 0 to 10 scale before and after each session and 24 hours after each session.

Patients also document how physically active they were per training week (activity levels A-C), notified in minutes per activity level. Level A consists of competitive sports activities, intensive enough to cause heavy sweating. Level B consists of endurance or muscle strengthening activities, intense enough to cause light sweating and faster breathing while still allowing one to talk, i.e. increased walking, running, cycling, swimming, cross-country skiing or muscle strength training. Level C consists of activities of daily living, i.e. walking, climbing stairs, housekeeping or garden work [25].

#### Knee school

The knee school was designed to educate participants about knee OA, the preparation phase before undergoing TKR and the acute rehabilitation phase afterwards. The concept of the knee school is adapted from the hip school, described by Klassbo [26]. The knee school is taught by an experienced and especially trained physical therapist over 3 individual or group sessions, one session per week, starting about 4 weeks before the operation. Knee school sessions are separately organised for participants of the intervention group and those of the control group to avoid contamination.

The content of the school includes information about anatomy of the knee joint and adjacent functional structures, recommended activities with prosthesis and postoperative pain management, and details on the postoperative rehabilitation phase. Didactical elements include models of the knee joint and the lower extremity, working sheets, photos and videos, hand outs, PowerPoint presentations and peer discussions.

#### Intervention group

Participants of the intervention group receive 8–24 sessions of the neuromuscular training program prior to TKR surgery depending on location of the waiting list for surgery. Participants also receive 3 sessions of the knee school starting about 4 weeks before surgery.

### Control group

Participants of the control group receive 3 sessions of the knee school, starting about 4 weeks before surgery.

#### Outcome measures

We include one objective (Chair Stand Test) and two patient-reported outcome measures (pain and function subscale of the KOOS questionnaire) in this trial to test the effect of the intervention on lower extremity function (see Table 1).

**Table 1:** Summary of measures to be collected

**Primary outcome measure**	**6**–**12 weeks pre**-**op**.	**1 week pre**-**op**.	**at discharge**	**6 weeks post**-**op**.	**3 months post**-**op**.	**1 year post**-**op**.
Chair Stand Test	X	X			X	
**Secondary outcome measure**						
Subscales pain and function of the KOOS	X	X		X	X	X
**Additional outcome measures**						
Muscle strength	X	X			X	
Knee-bending/30s	X	X			X	
Range of motion of the knee	X	X			X	
20m walk test	X	X			X	
Timed up and go	X	X			X	
Physical activity level	X	X		X	X	X
Adapted NHANES III	X	X		X	X	X
SF 36	X	X		X	X	X
EQ-5D	X	X		X	X	X
RAPT	X					
LOS			X			
Minutes of nursing care			X			

KOOS Knee injury and Osteoarthritis Outcome Score.

NHANES National Health And Nutrition Examination Survey.

SF 36 Short Form-36 health survey.

EQ-5D EuroQol – 5 dimensions.

RAPT Risk Assessment and Predictor Tool.

LOS Length of stay.

#### Primary outcome measure

The primary outcome is the Chair Stand Test, also known as the repeated sit-to-stand test. It is commonly used as a measure of lower extremity strength, balance and reaction time [27-29]. The subject is asked, to stand up from a chair without armrests to a fully erect standing position five times as quickly as possible without pushing off [30]. The time needed is measured by a stop watch in seconds. The Chair Stand Test is easy to perform in clinical practice and has shown excellent intra- and inter-rater reliability (Inter Class Correlation, 0.89 [31]. The Chair Stand Test was also found to predict disability across populations accurately [32].

#### Secondary outcome measure

Secondary outcomes are knee pain and function assessed by the KOOS questionnaire. The KOOS is a commonly used patient-reported outcome with overall acceptable psychometric properties to evaluate patients with knee injury and knee OA [33], including those having TKR [34]. KOOS holds 5 subscales with a total of 42 items: 1) pain, 2) other symptoms, 3) function in daily living (ADL), 4) function in sport and recreation and 5) knee-related quality of life. Since exercise training is aiming to improve function we are particularly interested in the KOOS ADL subscale for the functional outcome measure.

The German version of the KOOS is used in this trial [35]. Its user’s guide including scoring instructions, are available from http://www.koos.nu.

#### Additional outcome measures

##### Lower limb function

Isometric muscle strength of knee flexors and extensors, hip flexors, hip extensors, hip adductors and hip abductors are measured with a hand-held pull gauge [36,37]. The ability to alternate rapidly between concentric and eccentric work of the extensor muscles of the hip and knee is impaired in many patients with knee OA [38]. The ability of rapid alternation between concentric and eccentric function is measured using maximal number of knee-bending in 30 seconds, which is a valid and reliable test (ICC, 0.80) [24,39]. Range of motion is measured with a long-arm goniometer [40]. Walking speed is assessed with the 20m walk test (ICC, 0.93) [24], a reliable modification of the short walk test used in many epidemiological and clinical studies. The test measures the time it takes to walk 20 meters at the participant’s usual walking pace, along with the number of steps that they take to walk 20 meters [41]. Lower extremity mobility is further assessed with the Timed Up and Go test, which requires a person to rise from a stair, walk 4 meters, turn 180°, return to the chair and sit down [42].

##### Physical activity and health-related quality of life

Physical activity is measured by the SenseWear armband, a device for quantifying physical activity in daily life [43,44]. It collects the following data: energy expenditure, average MET’s, physical activity duration, steps per day and the physical activity distribution (sedentary, moderate, vigorous and very vigorous). In addition, physical activity is measured by 10 activity questions assessed and validated in NHANES III [45,46]. Health-related quality of life is measured by the generic questionnaire SF-36 [47,48]. General health status is measured by the EuroQoL (EQ-5D). The EQ-5D is used to complement the SF-36, allowing health economic evaluation and comparison to other knee OA populations [49].

##### Health service utilization

Discharge potential is measured by the Risk Assessment and Predictor Tool (RAPT), a 6 question tool which scores patients into low, middle or high risk for extended inpatient rehabilitation [23].

Length of Stay (LOS) and minutes of care by the nursing staff (including all activities delivered to the patients, e.g. mobilisation, washing, medication) are measured by LEP^®^, which is a tool used for workload management in nursing [50]. Both measurements are taken at discharge in the acute care hospital.

### Sample size calculation

The sample size calculation is based on the primary endpoint - the Chair Stand Test. We assume that the mean difference in change over time between groups is 7.3 seconds (corresponding to means of 8.3 and 1.0, respectively) and the common within-group standard deviation is 7.3. This effect was selected based on pilot data of an uncontrolled trial in knee OA patients [24], assuming that our control group (without exercise training) would not improve over time while awaiting TKR. It is also assumed that the effect size is reasonable, in the sense that an effect of this magnitude could be anticipated in this field of research. Alpha has been set at 0.05 and the power has been set at 0.9. In each group 25 patients are needed.

Assuming a drop-out rate of 12%, we will include 40 patients per group.

### Analysis plan

All analyses are based on intent-to-treat, including all randomized individuals who had at least 1 intervention session. We will use linear regression to evaluate the effect of our training program plus knee school against knee school alone for both the primary and secondary endpoints – after the training and at 3 months after surgery. The multivariate analyses for the primary endpoint (Chair Stand Test) and secondary endpoints (pain and function subscales of the KOOS) will control for baseline function of the respective endpoint, age in years, gender, and baseline body mass index. To account for the reduced exposure time to the training intervention for participants who were on a short waiting list or were less adherent we will perform subgroup analyses by training exposure groups (we will use the median as a cut-off for lower and higher intensity of the training program based on number of training sessions attended).

To evaluate a benefit of the training program on patient-reported pain and function across all time points, we will use repeated measures linear regression analyses controlling for baseline strength/function, time, age, gender, and body mass index.

Analyses will be conducted with R version 2.14.1 software. All P values are two-sided.

## Discussion

This randomized controlled trial will compare the effect of pre-operative neuromuscular training plus knee school with knee school alone on lower extremity function. The feasible training program aims to improve the activity-oriented outcome measures. The knee school serves both as an addition to the training as well as an attention control strategy in the control group. As an integral part of this trial, we assess adherence to the training intervention, which will allow per protocol analyses in addition to the primary intent-to-treat analyses.

OA is a key driver of disability at older age and is the fourth most common condition in older women and the eight most common in older men [6,51]. Given the demographic development with a rising segment of the senior population, and therefore improving functionality in individuals with OA is a public health target. Our trial aims to contribute important knowledge on whether to what extend a pre-operative training among patients on a waiting list for TKR will improve their functionality before and after surgery. The interest in the effect of pre-operative interventions has increased in the last decade, which is seen by the increasing number of publications. The evidence is up to now for different reasons moderate and a conclusive answer is not yet possible [12,52].

Our trial will also contribute to evidence-based guidelines to help physicians and patients make informed decisions [53]. With respect to patient functional status, the major predictor of patient functional status postoperatively is pre-operative status [54]. It is therefore important to test the benefit of pre-operative training in patients undergoing TKR and our trial could contribute to a definition of pre-operative treatment recommendations or guidelines.

## Abbreviations

TKR: Total Knee Replacement; OA: Osteoarthritis; RCT: Randomized Controlled Trial; KOOS: Knee Injury and Osteoarthritis Outcome Score; TJR: Total Joint Replacement; EULAR: European League Against Rheumatism; OARSI: Osteoarthritis Research Society International; RAPT: Risk Assessment and Predictor Tool; NEMEX: NEuroMuscular Exercise; ADL: Activities of daily living; MET: Metabolic Equivalent of Task; NHANES: National Health And Nutrition Examination Survey; SF-36: Short Form-36 health survey; EQ-5D: EuroQol - 5 dimensions; QoL: Quality of Life; LOS: Length of Stay.

## Competing interests

The authors declare that they have no competing interests.

## Authors’ contributions

EOH originated the idea of the study. EOH and HBF designed the trial protocol with assistance from EMR and RAB. EOH drafted the manuscript and the other authors revised it critically and corrected draft versions. All authors read and approved the final manuscript.

## Pre-publication history

The pre-publication history for this paper can be accessed here:

http://www.biomedcentral.com/1471-2474/14/157/prepub

## Supplementary Material

Additional file 1 NEMEX-TJR training program. Detailed description of the exercises. Click here for file
